# Different Fecal Microbiota in Hirschsprung's Patients With and Without Associated Enterocolitis

**DOI:** 10.3389/fmicb.2022.904758

**Published:** 2022-06-30

**Authors:** Alexis P. Arnaud, Ianis Cousin, Françoise Schmitt, Thierry Petit, Benoit Parmentier, Guillaume Levard, Guillaume Podevin, Audrey Guinot, Stéphan DeNapoli, Erik Hervieux, Valérie Flaum, Philine De Vries, Gwénaëlle Randuineau, Sandrine David-Le Gall, Sylvie Buffet-Bataillon, Gaëlle Boudry

**Affiliations:** ^1^Institut NuMeCan, INRAE, INSERM, Univ Rennes, Rennes-Saint-Gilles, France; ^2^Department of Pediatric Surgery, CHU Rennes, Univ Rennes, Rennes, France; ^3^Department of Pediatric Surgery, CHU Brest, Brest, France; ^4^Department of Pediatric Surgery, CHU Angers, Angers, France; ^5^Department of Pediatric Surgery, CHU Caen, Caen, France; ^6^Department of Pediatric Surgery, CHU Poitiers, Poitiers, France; ^7^Department of Pediatric Surgery, CHU Nantes, Nantes, France; ^8^Department of Pediatric Surgery, CHU Trousseau, APHP, Paris, France; ^9^Department of Infection Control and Prevention, CHU Rennes, Univ Rennes, Rennes, France

**Keywords:** Hirschsprung disease, Hirschsprung's associated enterocolitis (HAEC), fecal microbiota, proinflammatory bacteria, children

## Abstract

**Background and Objectives:**

Patients with Hirschsprung's disease are at risk of developing Hirschsprung-associated enterocolitis, especially in the first 2 years of life. The pathophysiology of this inflammatory disease remains unclear, and intestinal dysbiosis has been proposed in the last decade. The primary objective of this study was to evaluate in a large cohort if Hirschsprung-associated enterocolitis was associated with alterations of fecal bacterial composition compared with HD without enterocolitis in different age groups.

**Methods:**

We analyzed the fecal microbiota structure of 103 Hirschsprung patients from 3 months to 16 years of age, all of whom had completed definitive surgery for rectosigmoid Hirschsprung. 16S rRNA gene sequencing allowed us to compare the microbiota composition between Hirschsprung's disease patients with (HAEC group) or without enterocolitis (HD group) in different age groups (0–2, 2–6, 6–12, and 12–16 years).

**Results:**

Richness and diversity increased with age group but did not differ between HD and HAEC patients, irrespective of the age group. Relative abundance of Actinobacteria was lower in HAEC than in HD patients under 2 years of age (−66%, *P* = 0.045). Multivariate analysis by linear models (MaAsLin) considering sex, medications, birth mode, breast-feeding, and the Bristol stool scale, as well as surgery parameters, highlighted *Flavonifractor plautii* and *Eggerthella lenta*, as well as *Ruminococcus gnavus* group, as positively associated with Hirschsprung-associated enterocolitis in the 0–2 years age group.

**Conclusion:**

Hirschsprung-associated enterocolitis was associated with features of intestinal dysbiosis in infants (0–2 years) but not in older patients. This could explain the highest rate of enterocolitis in this age group.

**Clinical Trial Registration:**

https://clinicaltrials.gov/ct2/show/NCT02857205, MICROPRUNG, NCT02857205, 02/08/2016.

## Introduction

Hirschsprung's disease is a congenital disorder characterized by the absence of ganglionic cells in the myenteric and submucosal plexuses in the distal colon, causing neonatal functional bowel obstruction. This condition requires surgical resection of the aganglionic segment during the first months of life. Beyond surgical complications, patients are at risk of Hirschsprung-associated enterocolitis, whose incidence ranges from 4 to 54% with a mortality rate around 1% (Ruttenstock and Puri, 2010; Austin, 2012). Episodes of Hirschsprung-associated enterocolitis occur not only before surgery (5–57%) but also after the removal of the aganglionic bowel (2–35%) (Austin, 2012). Moreover, Hirschsprung-associated enterocolitis frequency decreases after 2 years of age (Menezes et al., 2006; Ruttenstock and Puri, 2010).

It is already known that Hirschsprung patients harbor a distinct fecal microbiota than healthy infants (Neuvonen et al., 2018). However, why some Hirschsprung patients develop Hirschsprung-associated enterocolitis but others do not remain unclear. Altered immune system and gut homeostasis maintenance mechanisms due to genetic susceptibility (Bachetti et al., 2021) or defects in epithelial cell barrier components (Gosain, 2016; Nakamura et al., 2018; Li et al., 2020) in Hirschsprung patients developing Hirschsprung-associated enterocolitis have been reported. A role of a specific gut microbiota composition in Hirschsprung-associated enterocolitis patients compared with non-enterocolitis ones, leading to altered barrier function and gut immune system development, is therefore likely in Hirschsprung-associated enterocolitis. Pioneer studies have suggested that bacterial overgrowth or the presence of specific bacterial or viral pathogens was associated with Hirschsprung-associated enterocolitis (Wilson-Storey et al., 1990; Hardy et al., 1993; Austin, 2012). However, no specific pathogen has been implicated in the etiology of Hirschsprung-associated enterocolitis so far. In the last decade, several reports using next-generation sequencing approaches compared the intestinal microbiota structure of Hirschsprung patients with or without Hirschsprung-associated enterocolitis in cross-sectional or prospective studies (De Filippo et al., 2010; Yan et al., 2014; Frykman et al., 2015; Demehri et al., 2016; Li et al., 2016; Neuvonen et al., 2018; Pini Prato et al., 2019; Tang et al., 2020; Arbizu et al., 2021). Although most of the studies found a difference in microbiota composition in Hirschsprung-associated enterocolitis compared with non-Hirschsprung-associated enterocolitis patients, these studies are often underpowered, with less than 20 patients enrolled. Interestingly, a recent prospective study identified a microbial signature of 21 operational taxonomic units (OTUs) in mucosal tissue sampled at surgery that would predict Hirschsprung-associated enterocolitis (Tang et al., 2020). However, the follow-up of patients was only until 3 years of age, hampering any conclusion on whether this altered microbiota structure lasts with age or not, which could explain the reduced incidence of Hirschsprung-associated enterocolitis after 2 years of age.

A comprehensive study of gut microbiota composition differences between Hirschsprung patients with or without a history of Hirschsprung-associated enterocolitis in a large cohort encompassing a large range of pediatric ages is needed to make a step forward in the understanding of the pathophysiology of Hirschsprung-associated enterocolitis. We therefore included 103 Hirschsprung patients aged 3 months to 16 years, in 7 different centers in France, all of whom had completed definitive surgery for rectosigmoid HD. 16S rRNA gene sequencing of fecal samples allowed us to compare the microbiota composition between Hirschsprung patients with (HAEC group) or without (HD group) Hirschsprung-associated enterocolitis in different age groups and to study whether differences in fecal microbiota structure between HAEC vs. HD patients were constant with age.

## Methods

### Patients

A multicenter (7) prospective study was conducted from May 2016 to January 2018 according to the principles expressed in the Declaration of Helsinki. The Ethics Review Board of Rennes University Hospital approved the research protocol (2015-A01317-42), and the study was registered on ClinicalTrials.gov (MICROPRUNG, NCT02857205). Inclusion criteria were patients below 16 years of age followed up for rectosigmoid Hirschsprung's disease attested by the pathology report, who had completed definitive surgery. Exclusion criteria were Hirschsprung's disease longer than the rectosigmoid, syndromic Hirschsprung's disease, Down syndrome, diverting stoma at the time of the study, and active Hirschsprung's associated enterocolitis at the time of stool collection. Written informed consent was obtained from parents during an outpatient clinic at each center. A detailed case report form was filled in during the outpatient clinic and completed with the patient's notes. Data included patients' characteristics, operative notes and surgical history, postoperative and long-term outcomes, details of Hirschsprung-associated enterocolitis episodes, neonatal diet, and medication, including antibiotics, probiotics, and prebiotics.

Patients were divided into two groups, namely, patients with a history of Hirschsprung-associated enterocolitis (HAEC) and patients without a history of Hirschsprung-associated enterocolitis (HD). Hirschsprung-associated enterocolitis was clinically defined as a distended abdomen associated with abdominal pain, diarrhea or smelly stools, and/or fever (Elhalaby et al., 1995). Patients were also arbitrarily grouped according to age at sampling: 0–2 years (period of time with an increased risk of Hirschsprung-associated enterocolitis), 2–6 years (potty training), 6–12 years, and 12–16 years (pubertal age).

### Functional Evaluation

Functional evaluation was based on the Krickenbeck International classification for postoperative results (Holschneider et al., 2005) and the Ann Arbor questionnaire (El-Sawaf et al., 2007) and was assessed in patients older than 3 years.

### Stool Sample, Fecal DNA Isolation, and Sequencing

After consent, patients were given a stool sampling kit following the recommendations of the International Human Microbiome Standards (IHMS) (Dore et al., 2015). Stool sampling was performed within 15 days after enrollment. Samples were sent by patients following the IHMS recommendations within 7 days of collection to the Biological Resources Center of Rennes University Hospital for storage at −80°C until analysis. At completion of the study, DNA was isolated from stool samples using the MagNA Pure LC DNA Isolation Kit III (Roche) following the manufacturer's instructions. The V3-V4 region of 16S rRNA gene was amplified and sequenced using Illumina Miseq technology as already described (Arnaud et al., 2020). Raw sequences were analyzed using the bioinformatic pipeline FROGS (Escudié et al., 2018) as already described (Arnaud et al., 2020). The dataset generated and analyzed during the current study are available in Data INRAE/Numecan repository, https://doi.org/10.15454/15RXTU.

### Statistics

Data are presented as mean ± standard error of the mean. Hirschsprung-associated enterocolitis frequency differences according to the factor studied was analyzed using a chi-square test. The Physloseq package was used for biostatistical process of microbiota sequencing data. The number of unique observed species and Shannon index (evenness of the species abundance distribution) were calculated and differences among groups were evaluated using Kruskal-Wallis tests. Beta-diversity was evaluated by calculating Jaccard, Bray-Curtis, unweighted, and weighted Unifrac distances between samples. Differences between groups were tested using PERMANOVA and Adonis function. Differences in relative abundances of the 4 major phyla among age groups were compared using the Kruskal-Wallis test and between Hirschsprung-associated enterocolitis status within an age group using the Mann-Whitney test. Finally, multivariate analysis by linear models analysis [MaAsLin (Mallick et al., 2021)] was run with defaults parameters to identify association of a specific microbial community member (operational taxonomic unit, OTU) with metadata. OTU count data were normalized and transformed according to methods available in the package (CSS normalization and log transformation). False discovery rate (FDR) values were calculated according to the Benjamini–Hochberg correction with respect to the age group and HAEC status. The model was additionally adjusted for sex, medications, probiotic use, birth mode, breast-feeding, Bristol stool score, age at surgery, and post-surgery complications. Only OTU associated with a Q-value < 0.01 and at least 40% of the samples (i.e., more than 41 patients out of the 103) were selected. Putative OTU identity was determined using NCBI Blastn++.

## Results

### Patients and Hirschsprung-Associated Enterocolitis History

**Figure 1:**
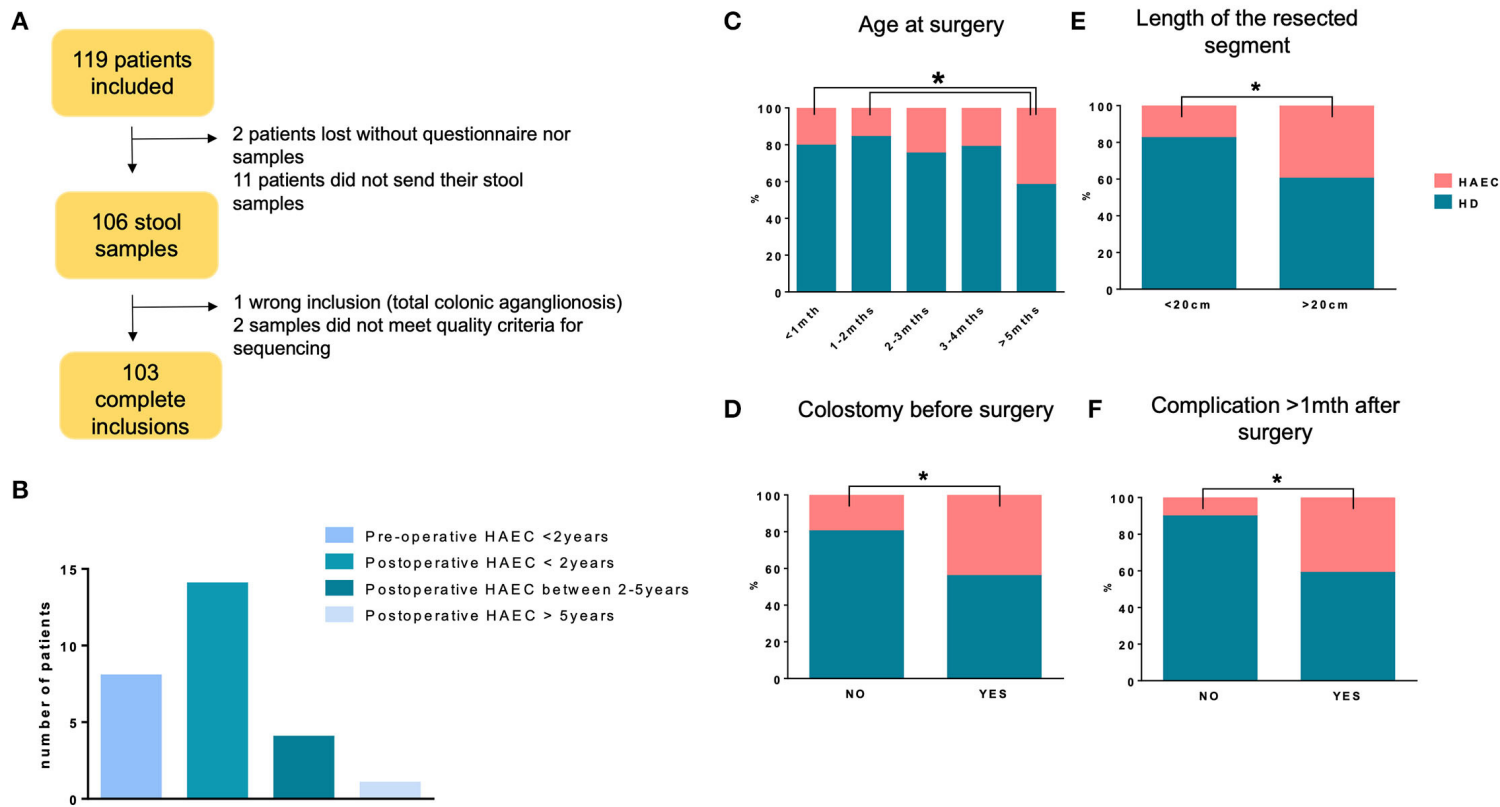
MICROPRUNG cohort characteristics. Flowchart of the cohort **(A)**, Hirschsprung-associated enterocolitis incidence as a function of age **(B)**, age at surgery **(C)**, existence of colostomy before surgery **(D)**, length of the resected segment **(E)**, and occurrence of complications more than 1 month after surgery **(F)**. *P < 0.05 for HAEC incidence.

A total of one hundred and nineteen patients agreed to participate to the research protocol. Inclusions were completed in 103 patients as shown in the flow chart (Figure 1A). Notably, 102 (99%) patients were operated on before 2 years of age with 75 (73%) before 3 months, 17 (16.5%) between 3 and 6 months, 8 (7.5%) between 6 and 12 months, and 2 (2%) between 12 and 24 months. In addition, twenty-five patients (24%) presented with a history of Hirschsprung-associated enterocolitis. Preoperative Hirschsprung-associated enterocolitis was observed in 9 cases (36% of total HAEC patients), among whom 1 had both preoperative and postoperative Hirschsprung-associated enterocolitis, both before 2 years of age. Of note, seventeen patients experienced postoperative Hirschsprung-associated enterocolitis: 13 patients presented with Hirschsprung-associated enterocolitis only before 2 years of age, 1 patient experienced Hirschsprung-associated enterocolitis before 2 years, and between 2 and 5 years of age, and 3 patients presented with Hirschsprung-associated enterocolitis after 2 years of age (Figure 1B). Patients with HAEC did not differ from HD patients in terms of sex ratio, history of Hirschsprung's disease in the family, birth weight, age at surgery, age at study, or bowel function evaluated by the Krickenbeck questionnaire [performed on patients older than 3 years (47 HD and 18 HAEC)] (Table 1). The global score of Ann Harbor, which evaluates the long-term complications post-pull-through for Hirschsprung's disease (continence, stooling pattern, enterocolitis), differed between HD and HAEC patients depending on age groups, with greater percentages of HAEC patients with complications (Table 1). The percentage of patients developing Hirschsprung-associated enterocolitis increased with increasing age at surgery (Figure 1C), preoperative colostomy (Figure 1D), intestinal resection length > 20 cm (Figure 1E), surgical complications, such as stenosis or hypertonic sphincter, developed more than 1 month after surgery (Figure 1F).

**Table 1:** Patients' characteristics.

	**All population**	**HD**	**HAEC**	***p*-value**
*N*	103	78 (76%)	25 (24%)	
Sex ratio male/female	4.1	3.5	7.3	0.4
History of Hirschsprung in the family	13	12	1	0.2
Birth weight (g)	3,425 ± 782	3,400 ± 785	3,565 ± 650	0.9
Age at surgery (days)	51 ± 67	48 ± 67	77 ± 90	0.3
Age at study (months)	59 ± 97	55 ± 91	66 ± 108	0.2
Age groups				
0–2 years	24	20	4	
2–6 years	36	26	10	
Years	26	21	5	
>12years	17	11	6	
Functional evaluation^a^				
Voluntary bowel movement	51 (77%)	38 (79%)	13 (72%)	0.5
Soiling symptoms	54 (83%)	38 (79%)	16 (89%)	0.7
Constipation	21 (32%)	16 (33%)	5 (28%)	0.7
Ann-Harbor score^*a*^				
2–6y				
Poor	–	0%	25%	<0.05
Fair	–	28%	0%	
Good	–	33%	25%	
Excellent	–	39%	50%	
6–12y				
Poor	–	0%	0%	
Fair	–	5%	0%	
Good	–	38%	44%	
Excellent	–	57%	56%	
12–16y				
Poor	–	0%	0%	
Fair	–	0%	17%	<0.05
Good	–	0%	50%	<0.05
Excellent	–	100%	33%	<0.05

^a^*Evaluation performed only on patients older than 3 years (47 HD and 18 HAEC)*.

### Factors Known to Affect Microbiota Composition Were Poorly Associated With Hirschsprung-Associated Enterocolitis Frequency

Several factors are known to greatly influence microbiota composition, especially in childhood. We therefore evaluated whether Hirschsprung-associated enterocolitis frequency differed according to these factors in our cohort.

**Figure 2:**
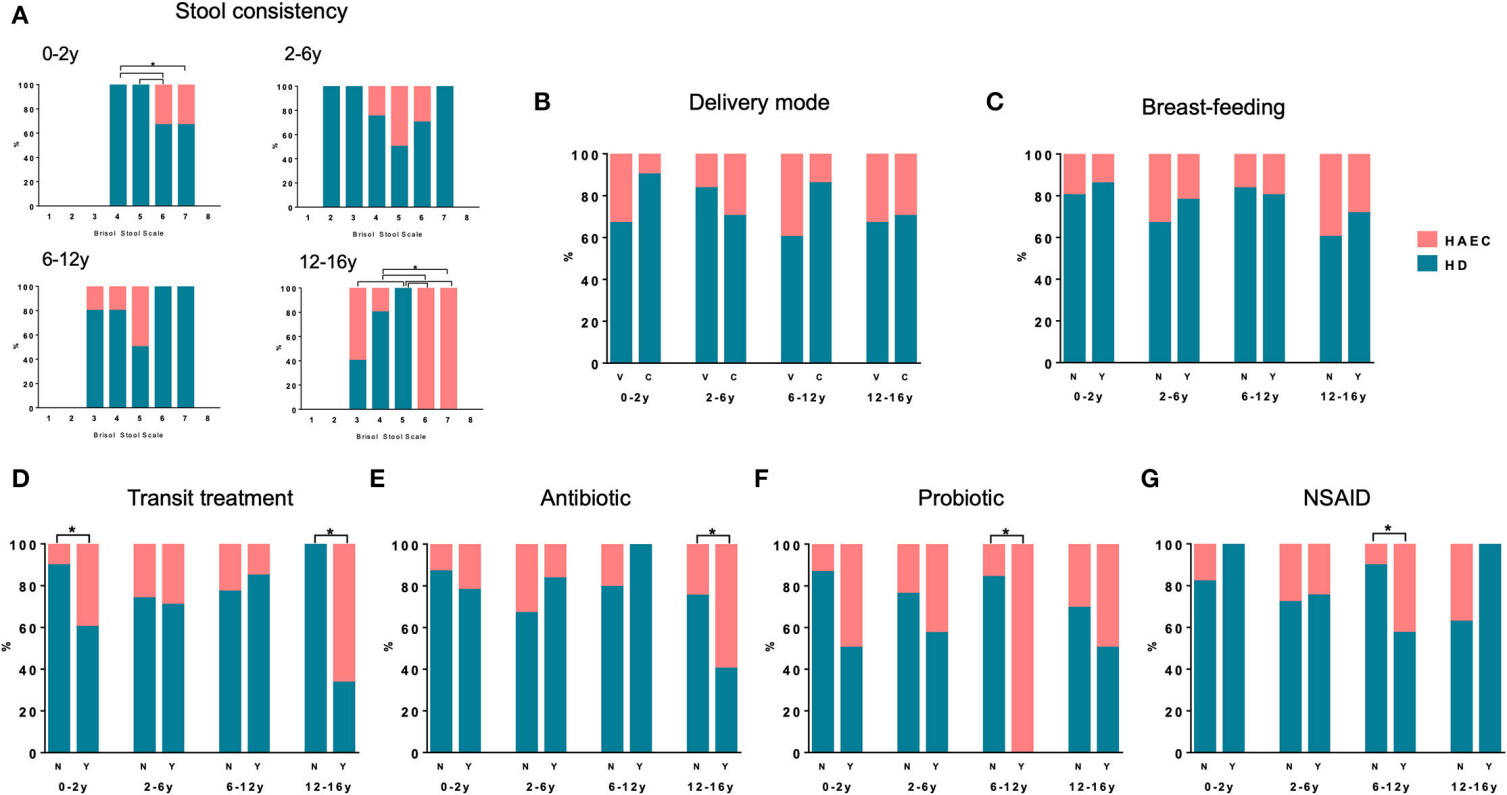
Hirschsprung-associated enterocolitis incidence and factors known to modulate gut microbiota. Hirschsprung-associated enterocolitis incidence as a function of stool consistency **(A)**, delivery mode **(B)**, breast-feeding **(C)**, treatment for transit **(D)**, antibiotic within the last 6 months **(E)**, probiotic treatment **(F)** or non-steroidal anti-inflammatory drug (NSAID) **(G)** in the different age groups. *P < 0.05 for Hirschsprung-associated enterocolitis incidence.

We first evaluated if stool consistency [(Bristol Stool Scale (BSS)] was associated with enterocolitis frequency in our cohort. At inclusion, the majority of the patients (81%) declared a BSS between 4 and 6. Stool consistency was not associated with greater frequency of Hirschsprung-associated enterocolitis in patients aged 2–6 or 6–12 years (Figure 2A). However, in the 0–2 year age group, greater BSS was associated with a greater frequency of HAEC (Figure 2A). Likewise, a lower or a greater BSS was associated with greater Hirschsprung-associated enterocolitis in the 12–16 year age group (Figure 2A). We next evaluated if neonatal conditions, such as delivery mode and breast-feeding, were associated with Hirschsprung-associated enterocolitis. In our cohort, 23% of the patients were born after C-section. Frequency of Hirschsprung-associated enterocolitis was not influenced by delivery mode (Figure 2B). Notably, 57% of the infants of our cohort had been breast-fed (exclusive or mixed breast-feeding). No difference in enterocolitis frequency was observed between breast-fed and non-breast-fed patients (Figure 2C).

We finally evaluated the impact of drugs on Hirschsprung-associated enterocolitis frequency. In our cohort, 43% of the patients were treated for transit disturbances at inclusion and thirty four (33%) patients currently used medication for bowel function. Hirschsprung-associated enterocolitis frequency was greater in 0–2 and 12–16 year old patients who were treated for transit (Figure 2D). Notably, 27% of the patients had received antibiotics for reasons other than Hirschsprung-associated enterocolitis in the 6 months preceding inclusion. No difference in Hirschsprung-associated enterocolitis frequency was observed between patients who had received antibiotics or not under 12 years, but the frequency of enterocolitis was greater in patients aged 12–16 years who had received antibiotics (Figure 2E). Probiotics and prebiotics have become popular over-the-counter treatments in patients suffering from gastrointestinal symptoms. In our cohort, 15% of the patients were taking probiotic treatment at the time of inclusion. Hirschsprung-associated enterocolitis frequency was greater in patients who were taking probiotics in the 6–12 year age group (Figure 2F). None of the patients reported prebiotic use in our cohort. Finally, 14% of the patients had taken non-steroidal anti-inflammatory drug (NSAID) in the last 6 months before inclusion. No difference in Hirschsprung-associated enterocolitis frequency was noted between patients taking NSAID or not except in the 6–12 years age group (Figure 2G).

### Fecal Microbiota of Patients Aged 0–2 Years Differed According to the HAEC Status

A total of 3,648,369 raw sequences were obtained after sequencing, which was reduced to 2,573,944 after filtration, clustering, and chimera removal. Of the total dataset, 12 phyla corresponding to 87 family and 192 genera were identified.

**Figure 3:**
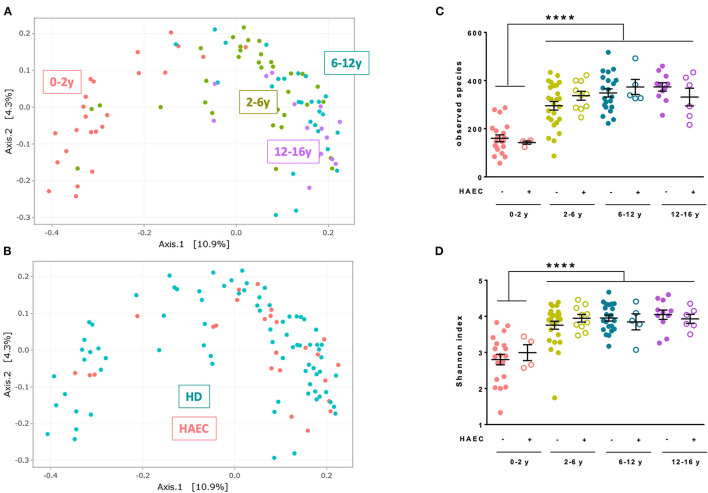
Characteristics of fecal microbiota of the MICROPRUNG cohort. Principal coordinates analysis representation of Jaccard distance between fecal microbiota highlighting age group **(A)** or Hirschsprung-associated enterocolitis status **(B)**. Number of observed species **(C)** and Shannon index **(D)** of the microbiota in function of age group and Hirschsprung-associated enterocolitis status. ****P < 0.0001.

Principal coordinates analysis with Jaccard distance showed a major effect of age on fecal microbiota composition, with clustering of the microbiota from patients aged 0–2 years compared with other age groups (age group effect, PERMANOVA *P* < 0.0001, Figure 3A). No clustering of HD vs. HAEC patients was noticed (HAEC effect, PERMANOVA *P* = 0.94, Figure 3B). Similar results were obtained for other distances (Bray-Curtis, Unweighted, and Weighed Unifrac, data not shown). Specific richness increased significantly with age with a lower richness in patients aged 0–2 years compared with older patients, with no difference observed between HD and HAEC patients, irrespective of the age group (Kruskal-Wallis adjusted *P*-values, 0–2 years vs. other age groups, *P* < 0.0001 for all comparisons, Figure 3C). Diversity, estimated by the Shannon index, increased with age with a lower diversity in patients aged 0–2 years compared with older patients but no difference between HD and HAEC patients (Kruskal-Wallis adjusted *P*-value, 0–2 years vs. other age groups *P* < 0.0001 for all comparisons, Figure 3D).

**Figure 4:**
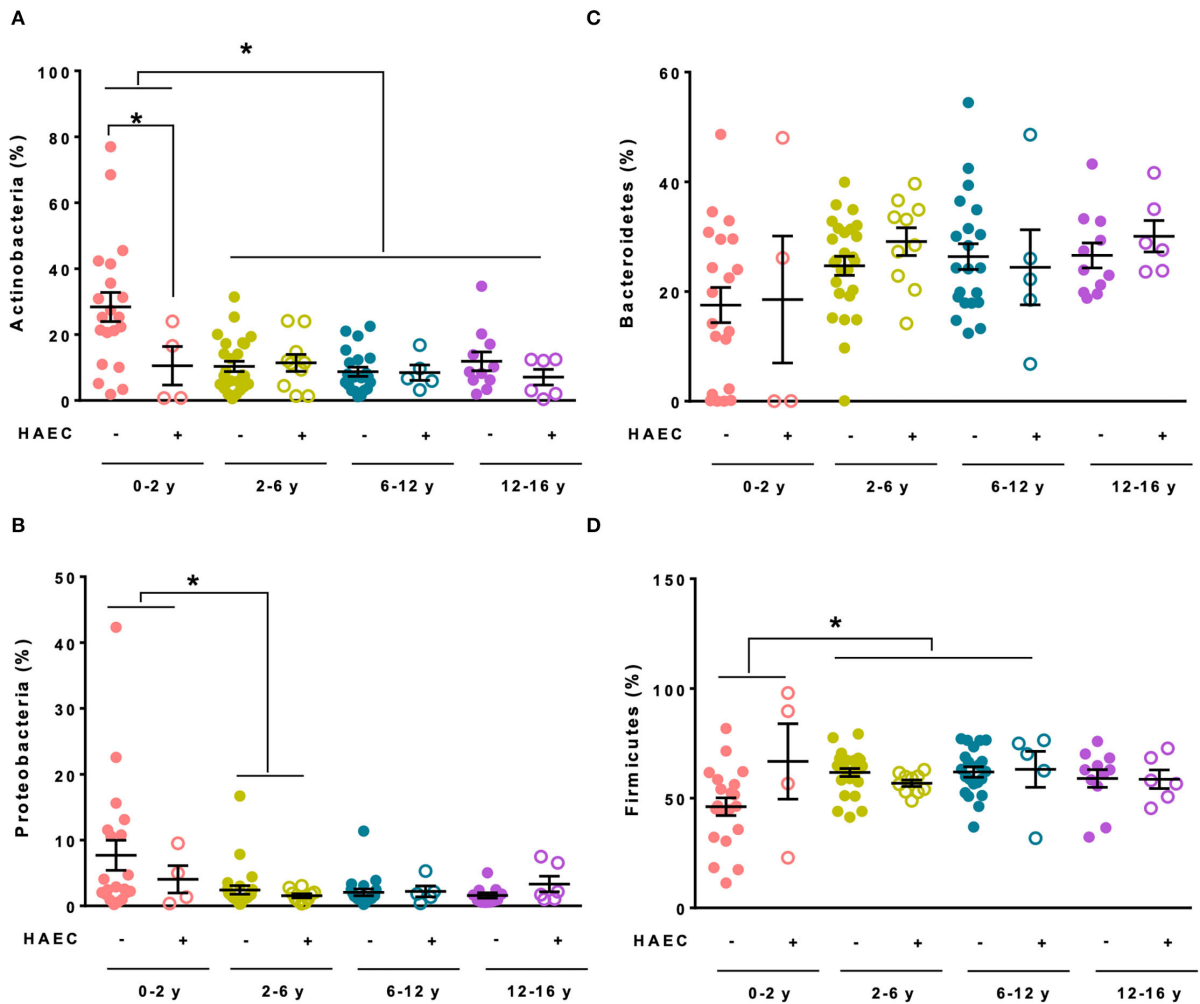
Main phyla relative abundances of fecal microbiota in the MICROPRUNG cohort. Relative abundance of Actinobacteria **(A)**, Proteobacteria **(B)**, Bacteroidetes **(C)**, and Firmicutes **(D)** in function of age group and Hirschsprung-associated enterocolitis status. *P < 0.05.

At the phylum level, several evolutions with age were noticed. The relative abundance of Actinobacteria (Kruskal-Wallis adjusted *P*-values: 0–2 vs. 2–6 years −33%, *P* = 0.013, 0–2 vs. 6–12 years −41%, *P* = 0.003, 0–2 vs. 12–16 years −36%, *P* = 0.037, Figure 4A) and Proteobacteria (Kruskal-Wallis adjusted *P*-values: 0–2 vs. 2–6 years −31%, *P* = 0.048, Figure 4B) decreased between groups 0–2 years and the other age groups, while that of Firmicutes (Kruskal-Wallis adjusted *P*-values: 0–2 vs. 2–6 years +58%, *P* = 0.055, 0–2 vs. 6–12 years +74%, *P* = 0.012, Figure 4D) increased with age groups. Relative abundance of Bacteroidetes (Figure 4C) did not vary significantly with age. Noteworthy, relative abundance of Actinobacteria was lower in HAEC than in HD patients in the 0–2 year age group (-66%, Mann-Whitney, *P* = 0.045, Figure 4A). This was primarily due to lower relative abundance of Bifidobacteriaceae family in HAEC patients, although not reaching significance (Mann-Whitney, −64%, *P* = 0.055, data not shown).

We next used MaAsLin, a multivariate statistical framework that finds associations between clinical metadata and microbial community abundance (Mallick et al., 2021). We constructed a first model that took into account age group and Hirschsprung-associated enterocolitis history (Table 2; Supplementary Table 1). We focused on the 0–2 year old patients presenting with Hirschsprung-associated enterocolitis, as this condition mostly happens in this age group. Eight OTUs that were significantly associated with this patient group, 7 from the Firmicutes phylum (*Flavonifractor plautii, Ruminococcus gnavus group, Blautia sp., Ruminococcae UBA1819, Dorea, Ruminococcus torques group*, and *Fusicatenibacter*) and one from Actinobacteria (*Eggerthella lenta*), were identified (Table 2). Because several factors have been associated with Hirschsprung-associated enterocolitis frequency, we constructed three other models (models 2, 3, and 4) that took into account these factors to evaluate the strength of association of these 8 OTUs with this patient group. The second model included patient sex, medications, birth mode, breast-feeding, and the Bristol stool scale. Among the 8 OTUs, only 3 (*Flavonifractor, Ruminococcus gnavus group*, and *Eggerthella*) remained significant in this model (Table 2). The third model took into account surgery parameters (age at surgery and complications later than 1 month post-surgery). The same 3 OTUs remained significantly associated with this patient group, as well as *Blautia* (Table 2). The fourth model considered all the abovementioned parameters. Two OTUs (*Flavonifractor* and *Eggerthella)* remained significantly associated with the 0–2 year old HAEC group (Table 2; Supplementary Figure 1). Two OTUs (*Subdoligranulum* and *Anaerotruncus*) were associated with the 2–6 year old HAEC group and one OTU (*Bifidobacterium*) with the 12–16 year old HAEC group in the first model but was no more associated in the three other complex models (Supplementary Table 1). No OTU was associated with the 6–12 year old HAEC group (Supplementary Table 1).

**Table 2:** Three OTUs were significantly associated with Hirschsprung-associated enterocolitis history in 0–2 year old patients, independent of other possible confounding factors.

	**Model 1**			**Model 2**			**Model 3**			**Model 4**		
	**Age** **×HAEC**			**Age** **×HAEC** **+** **medications**^**a**^ **+** **probiotic use** **+** **birth mode** **+** **breast-feeding** **+** **Bristol score**			**Age** **×HAEC** **+** **age at surgery** **+** **post-surgery complications**			**age** **×HAEC** **+** **sex** **+** **medications**^**a**^ **+** **probiotic use** **+** **birth mode** **+** **breast-feeding** **+** **Bristol score+** **age at surgery** **+** **post-surgery complications**		
**Taxonomy**	**Coef**	***P-*** **value**	***Q*** **-value**	**Coef**	***P*** **-value**	***Q*** **-value**	**Coef**	***P*** **-value**	***Q*** **-value**	**Coef**	***P*** **-value**	***Q*** **-value**
Firmicutes, Oscillospiraceae, *Flavonifractor plautii*	2.4 ×10^−2^	2.0 ×10^−10^	2.8 ×10^−7^	2.3 ×10^−2^	1.6 ×10^−8^	3.6 ×10^−5^	2.6 ×10^−2^	3.3 ×10^−10^	6.5 ×10^−7^	2.6 ×10^−2^	4.7 ×10^−6^	9.1 ×10^−3^
Actinobacteriota, Eggerthellaceae, *Eggerthella lenta*	5.4 ×10^−3^	2.6 ×10^−10^	2.8 ×10^−7^	5.2 ×10^−3^	1.7 ×10^−8^	3.6 ×10^−5^	6.0 ×10^−3^	3.0 ×10^−10^	6.5 ×10^−7^	6.1 ×10^−3^	8.6 ×10^−7^	2.4 ×10^−3^
Firmicutes, Lachnospiraceae, *Ruminococcus gnavus group*	1.7 ×10^−3^	1.3 ×10^−6^	5.9 ×10^−3^	1.7 ×10^−3^	8.8 ×10^−6^	5.9 ×10^−3^	1.7 ×10^−3^	1.6 ×10^−5^	5.9 ×10^−3^	–	–	–
Firmicutes, Lachnospiraceae, *Blautia*	7.5 ×10^−3^	1.3 ×10^−5^	1.7 ×10^−3^	^−^	–	–	8.6 ×10^−3^	7.7 ×10^−6^	4.0 ×10^−3^	–	–	–
Firmicutes, Ruminococcacea, *UBA1819*	1.5 ×10^−2^	1.5 ×10^−5^	1.9 ×10^−3^	–	–	–	–	–	–	–	–	–
Firmicutes, Lachnospiraceae, *Dorea*	1.3 ×10^−3^	5.0 ×10^−5^	4.1 ×10^−3^	–	–	–	–	–	–	–	–	–
Firmicutes, Lachnospiraceae, *Ruminococcus torques group*	8.5 ×10^−3^	2.3 ×10^−5^	2.2 ×10^−3^	^−^	^−^	–	–	–	–	–	–	–
Firmicutes, Lachnospiraceae, *Fusicatenibacter saccharivorans*	4.1 ×10^−2^	6.9 ×10^−5^	4.8 ×10^−3^	–	–	–	–	–	–	–	–	–

^a^*Antibiotics, transit treatment, Coef= model coefficient value (effect size) for that particular OTU, i.e., contrast between 0–2 year old HAEC group and 0–2 year old HD group taken as reference, P-value=nominal significance of the association in the model, Q-value=corrected significance of the association based on the whole dataset*.

*MaAsLin results for association of OTU with 0–2 year old HAEC patients. Only OTU associated with the 0–2 year old HAEC patient group with a Q-value > 0.01 and present in at least 40% of the samples (i.e., more than 41 patients out of the 103) were selected*.

## Discussion

Intestinal dysbiosis has been incriminated in many diseases, especially inflammatory ones (Petersen and Round, 2014). Hirschsprung's disease patients harbor a different intestinal microbiota composition than healthy patients (Neuvonen et al., 2018). Previous studies hypothesized that patients with Hirschsprung-associated enterocolitis harbor a further different microbiota compared with Hirschsprung's disease patients without enterocolitis, especially in the two first years of life. Indeed, Li et al. observed that patients with active Hirschsprung-associated enterocolitis or in remission had more similar microbiota composition, irrespective of Hirschsprung-associated enterocolitis activity and symptoms, than Hirschsprung patients without enterocolitis (Li et al., 2016). However, the cohort was very small (3 enterocolitis patients in remission, 8 patients with active enterocolitis, and 2 Hirschsprung patients without enterocolitis). For this reason, we wanted to compare gut microbiota composition between Hirschsprung patients with or without a history of Hirschsprung-associated enterocolitis in a large cohort encompassing a large range of pediatric ages. In our study enrolling 103 patients, fecal microbiota composition using the Jaccard between-samples distances or other metrics clearly clustered patients upon age but not upon Hirschsprung-associated enterocolitis status. This is in accordance with the relative stabilization of the microbiota around 2–3 years of age (Rodríguez et al., 2015). Nonetheless, Actinobacteria relative abundance was lower in HAEC patients under 2 years of age compared with HD patients. Strikingly, reduced abundance of Actinobacteria is a hallmark of microbiota maturation with postnatal age (Yatsunenko et al., 2012), suggesting that HAEC patients aged 0–2 years had a mature fecal microbiota despite their age. Moreover, MaAsLin analysis identified *Flavonifractor plautii* and *Eggerthella lenta*, and to a lesser extent *Ruminococcus gnavus*, as it was not significantly associated with the group in the final model, as strongly associated with 0–2 year old HAEC patients. A tendency for increased abundance of the genus *Ruminococcus* in HAEC patients compared with HD had already been observed in a previous study (Demehri et al., 2016), but no report of altered abundance of *Flavonifractor plautii* and *Eggerthella lenta* has been described so far. These three genera were found, among 40 others species, in a core microbiota associated with poor health in a multi-study integration of human stool metagenomes (Gupta et al., 2020). *Ruminococcus gnavus* and *Flavonifractor plautii* are associated with inflammatory bowel disease or colorectal cancer (Hall et al., 2017; Ai et al., 2019; Gupta et al., 2019; Li et al., 2021). *Eggerthella lenta* exacerbates colitis in murine models (Alexander et al., 2019). Thus, although *Flavonifractor plautii* has also been associated with improved gut inflammation in animal models (Mikami et al., 2021), these three genera seem to be inflammatory-disease associated bacteria.

The difference observed in fecal microbiota could have been due to antibiotic or probiotic treatment of Hirschsprung-associated enterocolitis patients, as they all received antibiotics during enterocolitis episode and the proportions of patients receiving probiotics was significantly greater in HAEC than in HD patients. Our MaAsLin analysis took into account these parameters, as well as other factors known to influence microbiota composition and/or Hirschsprung-associated enterocolitis incidence in our cohort or others. This not only includes delivery mode and Bristol stool scale but also surgery parameters, such as age at surgery or surgery complications. To the best of our knowledge, our study is the first to use numerous parameters to control for fecal microbiota difference, which strengthen the results. Importantly, we took into account breast-feeding, as this factor has recently been shown to be protective against Hirschsprung-associated enterocolitis in Hirschsprung's disease patients (Tang et al., 2020). In our cohort, this factor did not influence enterocolitis incidence. However, exclusive breast-feeding was not recorded and only exclusive or mixed vs. no breast-feeding at all was compared.

One strength of our study is the size of our cohort. Several studies strongly suggested dysbiosis in Hirschsprung-associated enterocolitis patients (De Filippo et al., 2010; Yan et al., 2014; Frykman et al., 2015; Demehri et al., 2016; Li et al., 2016; Neuvonen et al., 2018; Pini Prato et al., 2019; Tang et al., 2020; Arbizu et al., 2021). However, the difficulty to enroll patients with a disease, such as Hirschsprung's disease, largely underpowered these studies, whereas robust biological conclusion in microbiome studies requires well-powered cohort studies (Debelius et al., 2016). Calculation of sample size and power is challenging in microbiota studies due to the uniqueness of microbiota data and of the metrics used to describe community structure (richness, diversity indexes, abundances at the phylum, family, or species abundance). No “clinical” range in which these features should fall to be considered as pathologic or not has been established yet. Moreover, the intestinal microbiota ecosystem is strongly influenced by environmental and internal factors leading to a high within and between-individual variability (Flint, 2020). Here, we designed a cross-sectional multicenter study enrolling more than 100 patients. This sample size was the best compromise between study power and capacity to recruit patients. Moreover, sample collection and storage were standardized, and samples were processed together using the same reagents to avoid any technical bias. Nonetheless, our study might still be underpowered by the fact that we classified our patients in different age groups, leading to 18 to 37 patients per group and dissimilar distribution of HD and HAEC patients within each group (16% to 40% of HAEC). Even if we focused on the 0–2 year age group in our result description; a different analysis, especially MaAsLin analysis, was performed with the whole dataset (i.e., the 103 patients) and not only with the 0–2 year old patients. Thus, our study design enabled us to evaluate differences in microbiota composition of HD vs. HAEC patients within age groups and the changes with age.

Fecal microbiota composition differed between HD and HAEC patients before 2 years of age but not after, suggesting that microbiota composition is involved in Hirschsprung-associated enterocolitis pathophysiology.

One limitation of our study is the definition of Hirschsprung-associated enterocolitis. We decided to use the clinical definition described by Elhalaby et al. (1995) to find the criteria described in the patients notes retrospectively. A more recent Hirschsprung-associated enterocolitis scoring system (clinical, biological, and radiological criteria) has been described by Pastor et al. (2009) but is not routinely used in clinic and is very scarce in published studies (Frykman et al., 2017). This score was then validated by Frykman et al. (2017) with patients' data and used in few recent studies (Dore et al., 2019; Gunadi et al., 2020; Luzman et al., 2021). However, all these studies were retrospective, and the score still lacks an external validation with a prospective cohort as conceded by the authors (Frykman et al., 2017; Gunadi et al., 2020; Luzman et al., 2021).

Physicians dealing with Hirschsprung's disease patients with associated enterocolitis are waiting for a personalized therapy. The future is therefore to prevent or treat Hirschsprung-associated enterocolitis by targeting specific bacteria suspected to be responsible for these inflammatory episodes. Our study gives promising results in that way and requires confirmation with a further study.

In conclusion, our cross-sectional large cohort study confirmed our initial hypothesis of altered fecal microbiota composition in young Hirschsprung-associated enterocolitis patients which disappears with age. We also highlighted three genera associated with Hirschsprung-associated enterocolitis in young patients. Modulation of the intestinal microbiota, therefore, constitutes a promising strategy to prevent or treat Hirschsprung-associated enterocolitis.

## Data Availability Statement

The datasets presented in this study can be found in online repositories. The names of the repository/repositories and accession number(s) can be found below: https://data.inrae.fr/dataverse/numecan, https://doi.org/10.15454/15RXTU.

## Ethics Statement

The studies involving human participants were reviewed and approved by the Ethics Review Board of Rennes University Hospital. Written informed consent to participate in this study was provided by the participants' legal guardian/next of kin.

## Author Contributions

AA conceptualized and designed the study, collected data, drafted the initial manuscript, and reviewed and revised the manuscript. SD conceptualized and designed the study. IC, FS, TP, BP, GL, GP, AG, SD, EH, VF, and PD collected data and reviewed and revised the manuscript. SB-B and GR analyzed data and reviewed and revised the manuscript. GB conceptualized and designed the study, drafted the initial manuscript, and reviewed and revised the manuscript. All authors approved the final manuscript as submitted and agreed to be accountable for the content of the work.

## Funding

The study was funded by Rennes University Hospital (CORECT 2015).

## Conflict of Interest

The authors declare that the research was conducted in the absence of any commercial or financial relationships that could be construed as a potential conflict of interest.

## Publisher's Note

All claims expressed in this article are solely those of the authors and do not necessarily represent those of their affiliated organizations, or those of the publisher, the editors and the reviewers. Any product that may be evaluated in this article, or claim that may be made by its manufacturer, is not guaranteed or endorsed by the publisher.

## Abbreviations

BSS: Bristol Stool Scale
HD: Hirschsprung disease
HAEC: Hirschsprung disease associated enterocolitis
IHMS: International Human Microbiome Standards
MaAsLin: multivariate analysis by linear models
OTU: operational taxonomic unit.
