# Supplementary material for: Different Fecal Microbiota in Hirschsprung's Patients With and Without Associated Enterocolitis
Source: Front Microbiol. 2022 Jun 30;13:904758. doi: 10.3389/fmicb.2022.904758 (PMC9279138; doi:10.3389/fmicb.2022.904758)
Supplement: Supplementary file 1 [file Table_1.DOCX]

**Supplementary Table 1: List of OTUs significantly associated with the different subgroups in the different MaAsLin models.**

|  | **Model 1** | | | **Model 2** | | | **Model 3** | | | **Model 4** | | |
| --- | --- | --- | --- | --- | --- | --- | --- | --- | --- | --- | --- | --- |
|  | age x HAEC | | | age x HAEC + medications^1^ + probiotic use + birth mode + breast-feeding + Bristol score | | | age x HAEC + age at surgery + post-surgery complications | | | age x HAEC + medications^1^ + probiotic use + birth mode + breast-feeding + Bristol score+ age at surgery + post-surgery complications | | |
| **Taxonomy** | Coef | P-value | Q-value | Coef | P-value | Q-value | Coef | P-value | Q-value | Coef | P-value | Q-value |
| 0-2y HD | | | | | | | | | | | | |
| - | - | - | - | - | - | - | - | - | - | - | - | - |
| 2-6y HD | | | | | | | | | | | | |
| Actinobacteriota, Bifidobacteriaceae, *Bifidobacterium* | -1.5x  10^-1^ | 1.0x  10^-7^ | 4.2x  10^-4^ | -1.5x  10^-1^ | 1.0x  10^-5^ | 5.9x  10^-3^ | -1.7x  10^-1^ | 5.6x  10^-7^ | 4.4x  10^-4^ | - | - | - |
| 2-6y HAEC | | | | | | | | | | | | |
| Firmicutes, Ruminococcacea, *Subdoligranulum* | 2.9x  10^-2^ | 7.0x  10^-5^ | 4.2x  10^-3^ | - | - | - | - | - | - | - | - | - |
| Firmicutes, Ruminococcacea, *Anaerotruncus* | 5.9x  10^-4^ | 3.9x  10^-6^ | 8.2x  10^-4^ | - | - | - | - | - | - | - | - | - |
| 6-12y HD | | | | | | | | | | | | |
| Actinobacteriota, Bifidobacteriaceae, *Bifidobacterium* | -1.5x  10^-1^ | 2.5x  10^-7^ | 1.3x  10^-4^ | - | - | - | -1.8x  10^-1^ | 2.4x  10^-7^ | 2.3x  10^-4^ | - | - | - |
| Bacteroidota, Rikenellaceae, *Alistipes* | 4.2x  10^-2^ | 5.6x  10^-5^ | 4.2x  10^-3^ | - | - | - | 4.7x  10^-2^ | 3.4x  10^-5^ | 9.6x  10^-3^ | - | - | - |
| Firmicutes, Oscillospiraceae, UCG.002 | 4.2x  10^-2^ | 2.8x 10^-6^ | 7.4x  10^-4^ | - | - | - | 4.2x  10^-2^ | 1.0x  10^-5^ | 4.0x  10^-3^ | - | - | - |
| Firmicutes, Lachnospiraceae, *Eubacterium ventriosum* group | 3.2x  10^-3^ | 1.6x  10^-5^ | 1.9x  10^-3^ | - | - | - | - | - | - | - | - | - |
| 6-12y HAEC |  |  |  |  |  |  |  |  |  |  |  |  |
| - | - | - | - | - | - | - | - | - | - | - | - | - |
| 12-16y HD | | | | | | | | | | | | |
| Firmicutes, Ruminococcaceae, *Subdoligranulum* | 3.3x  10^-2^ | 5.9x  10^-6^ | 1.0x  10^-3^ | 3.6x  10^-2^ | 1.1x  10^-5^ | 5.9x  10^-3^ | 3.6x  10^-2^ | 1.0x  10^-5^ | 4.0x  10^-3^ | - | - | - |
| Firmicutes, Oscillospiraceae, UCG.002 | 6.7x  10^-2^ | 3.5x  10^-9^ | 2.5x  10*-6* | 6.3x  10^-2^ | 4.5x  10^-7^ | 6.7x  10^-2^ | 7.2x  10^-2^ | 5.5x  10^-9^ | 7.3x  10^-6^ | - | - | - |
| Actinobacteriota, Bifidobacteriaceae, *Bifidobacterium* | -1.4x  10^-1^ | 1.3x  10^-4^ | 8.3x  10^-3^ | - | - | - | -1.8x  10^-1^ | 1.8x  10^-5^ | 6.0x  10^-3^ | - | - | - |
| Firmicutes, Lachnospiraceae, *Eubacterium hallii* group | 1.3x  10^-3^ | 2.6x  10^-5^ | 2.3x  10^-3^ | - | - | - | 1.5x  10^-3^ | 1.0x  10^-5^ | 4.0x  10^-3^ | - | - | - |
| Firmicutes, Ruminococcaceae, *Negativibacillus* | 5.3x  10^-3^ | 2.1x  10^-5^ | 2.2x  10^-3^ | 2.0x  10^-3^ | 1.3x  10^-5^ | 5.9x  10^-3^ | - | - | - | - | - | - |
| Firmicutes, Oscillospiraceae, UCG.003 | 3.1x  10^-3^ | 1.5x  10^-4^ | 8.8x  10^-3^ | - | - | - | - | - | - | - | - | - |
| Firmicutes, Butyricicoccaceae, UCG.009 | 5.5x  10^-4^ | 3.1x  10^-5^ | 2.7x  10^-3^ | - | - | - | - | - | - | - | - | - |
| 12-16y HAEC | | | | | | | | | | | | |
| Actinobacteriota, Bifidobacteriaceae,*Bifidobacterium* | -1.8x  10^-1^ | 9.5x  10^-5^ | 6.1x  10^-3^ | - | - | - | - | - | - | - | - | - |

^1^ antibiotics, transit treatment, Coef= model coefficient value (effect size) for that particular OTU, i.e. contrast between the studied group and 0-2y HD group taken as reference, P-value=nominal significance of the association in the model, Q-value=corrected significance of the association based on the whole dataset.

Only OTU with a Q-value < 0.01 and present in at least 40% of the samples (i.e. more than 41 patients out of the 103) were selected.
