# Supplementary figures and images for: Different Fecal Microbiota in Hirschsprung's Patients With and Without Associated Enterocolitis
Source: Front Microbiol. 2022 Jun 30;13:904758. doi: 10.3389/fmicb.2022.904758 (PMC9279138; doi:10.3389/fmicb.2022.904758)

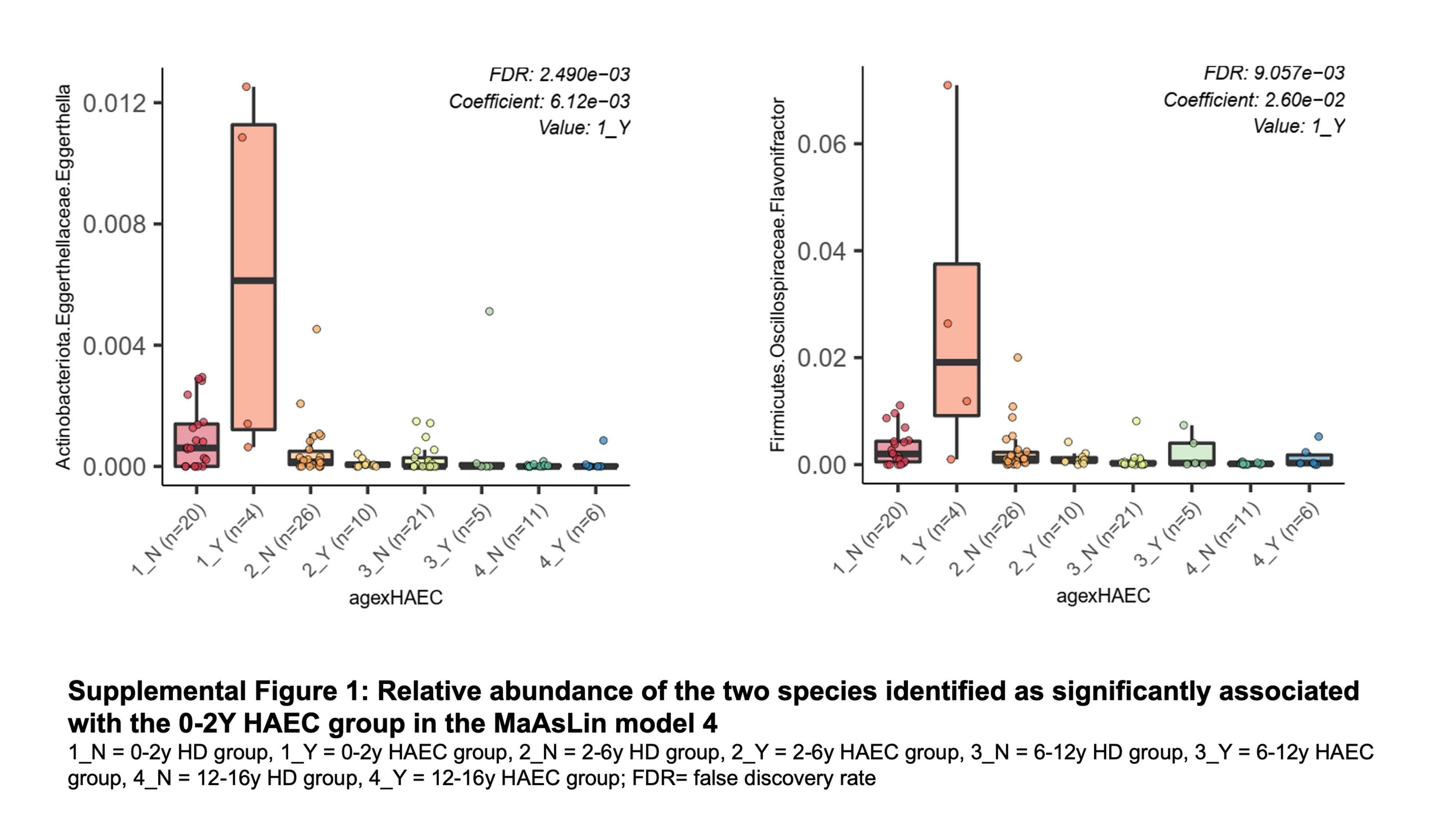

Supplement: Supplementary file 2 [file Image_1.TIFF]
